# Cytomorphological findings in diagnosis of Warthin tumor

**DOI:** 10.3906/sag-1901-215

**Published:** 2020-02-13

**Authors:** Fatma Fulya KÖYBAŞIOĞLU, Binnur ÖNAL, Ünsal HAN, Ayşegül ADABAĞ, Ahmet ŞAHPAZ

**Affiliations:** 1 Department of Pathology, Faculty of Medicine, Yüksek İhtisas University, Ankara Turkey; 2 Department of Pathology, Faculty of Medicine, Düzce University, Düzce Turkey; 3 Department of Pathology, Dışkapı Yıldırım Beyazıt Training and Research Hospital, Ankara Turkey; 4 Department of Histopathology, Forensic Medicine Institution, Erzurum Turkey

**Keywords:** Warthin tumor, fine needle aspiration cytology, diagnosis

## Abstract

**Background/aim:**

To define the cytomorphologic findings leading to difficulties in diagnosis of Warthin tumors (WTs).

**Materials and methods:**

Forty-eight histopathologically diagnosed WT patients who had fine needle aspiration cytology preoperatively were reevaluated for defining the presence or absence of lymphocytes, oncocytic cell layer, oncocytic cell papillae, granular debris background, mucoid background, macrophages, polymorphonuclear cells, mast cells, squamous-like cells, atypical vacuolated cytoplasmic cells, and giant cells.

**Results:**

Forty-seven tumors were in the parotid gland and one in the submandibular gland. There were 37 (77%) male and 11 (23%) female patients. Cytopathologically in 36 patients the diagnosis was benign neoplasm (WT); in 6, other benign entities; and in 6, suspicious for malignancy. The main characteristic cytomorphologic features of WTs were as follows: 92% lymphoid cells, 83% oncocytic cell layers, and 67% granular debris background. These percentages were 67%, 17%, and 17% in the benign cytology group and 67%, 50%, and 17% in the suspicious for malignancy group, respectively.

**Conclusion:**

Absence or lack of main features of WTs with or without presence of squamous-like cells, vacuolated cytoplasmic cells, and inflammatory reaction may cause diagnostic dilemma. The presence of the mast cells accompanied by epithelial tissue was striking for WT diagnosis.

## 1. Introduction

Warthin tumor (WT) is the second most common benign neoplasm of the parotid gland [1]. It is located almost exclusively in the parotid gland and may occur bilaterally or as multiple lesions [2,3]. Morphologically the tumors are usually cystic, and cysts are composed of lymphoid stroma lined by double rows of epithelial cells with oncocytic papillae [1]. Fine needle aspiration cytology (FNAC) is a simple and noninvasive method for preoperative diagnosis of the salivary gland tumors, and the diagnosis of WT is easy in the presence of the typical cytomorphological findings. The predominant features of the tumor are the cellular elements and cellular debris in the background, which consists of proteinaceous substrates. The cellular elements are scattered lymphoid cells and oncocytic cells with single and multiple layers. The oncocytic cells have abundant granular cytoplasm, round nuclei, and nucleoli. Squamous, mucoid, and mast cells can also be seen in slides [4,5]. Despite the well documented histopathologic picture, various cytomorphologic appearances of WTs in FNAC may lead to wrong cytopathologic interpretations. Our study aimed to define the cytomorphologic findings causing difficulties in the diagnosis of WT.

## 2. Materials and methods

In this retrospective study, we searched saved data of the Pathology Department of Dışkapı Yıldırım Beyazıt Training and Research Hospital between 2000 and 2015, and 48 histopathologically confirmed WT cases were enrolled. All patients had FNAC preoperatively. Air dried smears were stained by May Grünwald Giemsa (MGG) and were fixed with alcohol and were stained by Papanicolaou (PAP) stain and hematoxylin and eosin (H&E). Presence or absence of cytomorphologic findings such as lymphocytes, oncocytic cell layer, papillae with oncocytic cells, granular debris background, mucoid background, macrophages, polymorphonuclear (PMN) cells, mast cells, squamous-like cells, atypical cells with vacuolated cytoplasm, and giant cells were reevaluated. Cytomorphologic results were categorized as benign neoplasm (WT), other benign entities, and suspicious for malignancy. 

Results were analyzed by the IBM SPSS 20 statistical analysis program (Armonk, NY, USA). Data were presented as mean, standard deviation, median, minimum, maximum, percentage, and number. The normal distribution of continuous variables was analyzed by Shapiro–Wilk test. In the 2 × 2 comparisons between categorical variables, the Pearson chi-square test was used if the expected value was >5, the Yates chi-square test if the expected value was between 3 and 5, and Fisher’s exact test if the expected value was <3. Pearson’s chi-square test was used in cases where the expected value was >5 and the Fisher–Freeman–Halton test was used in cases where the expected value was <5. Statistical significance level was taken as P < 0.05.

Required permission for the study has been obtained from the relevant authority.

## 3. Results

Forty-seven tumors were in the parotid gland and one in submandibular gland. There were 37 (77%) male and 11 (23%) female patients and no bilateral tumor was seen. Median age was 56 years (ranging from 23 and 78). Cytopathologic examination results were benign neoplasm and other benign entities in 42 of 48 patients. The other 6 cases were suspicious for malignancy. In 36 of 42 patients with benign neoplasm and other benign entities, WT was diagnosed correctly. In 6 other patients with benign entities, the diagnoses were 1 oncocytoma, 1 chronic sialadenitis, 1 inflamed cyst, 1 mucocele, and 2 intraparotid lymphoid tissue. Permanent section results of the 6 cases suspicious for malignancy were reported as WT.

**Figure 1:**
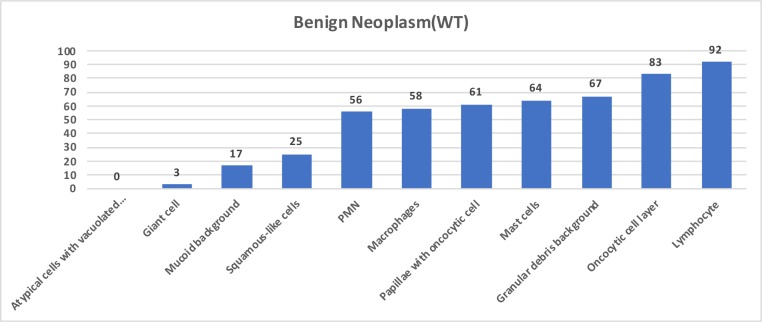
Graphic form of benign neoplasm (Warthin tumor) cytomorphologic features as percentage.

The cytomorphologic findings of 36 WT tumors were detailed as lymphocyte in 33 (92%), oncocytic cell layer in 30 (83%), granular debris background in 24 (67%), mast cells in 23 (64%), papillae with oncocytic cells in 22 (61%), macrophages in 21 (58%), PMN cells in 20 (56%), squamous-like epithelial cell in 9 (25%), and mucoid background in 6 (17%) cases (Table; Figure 1). In this group, oncocytic cell layer (P = 0.002), granular debris background (P = 0.031), mucoid background (P = 0.014), and mast cells (P = 0.005) were found statistically significant in the cytomorphologic diagnosis of WT.

**Table 1:** Percentage of WT cases reflecting cytomorphologic features.

	Benign neoplasm (WT)in FNAC (n = 36)	Other benign entitiesin FNAC (n = 6)	Suspicious for malignancyin FNAC (n = 6)	
Cytomorphologic features	(%) (n)	(%) (n)	(%) (n)	P-value
Lymphocytes	92 (33)	67 (4)	67 (4)	0.087
Oncocytic cell layer	83 (30)	17 (1)	50 (3)	0.002*
Granular debris background	67 (24)	17 (1)	83 (5)	0.031*
Papillae with oncocytic cell	61 (22)	17 (1)	17 (1)	0.052
Mucoid background	17 (6)	67 (4)	50 (3)	0.014*
Macrophages	58 (21)	33 (2)	33 (2)	0.384
PMN cells	56 (20)	33 (2)	33 (2)	0.441
Mast cells	64 (23)	-	33 (2)	0.005*
Squamous-like cells	25 (9)	-	83 (5)	0.005*
Atypical cells with vacuolated cytoplasm	-	-	17 (1)	0.250
Giant cells	3 (1)	-	-	1.000

WT: Warthin tumor, FNAC: Fine needle aspiration cytology, PMN: polymorphonuclear. *: P < 0.05.

**Figure 2:**
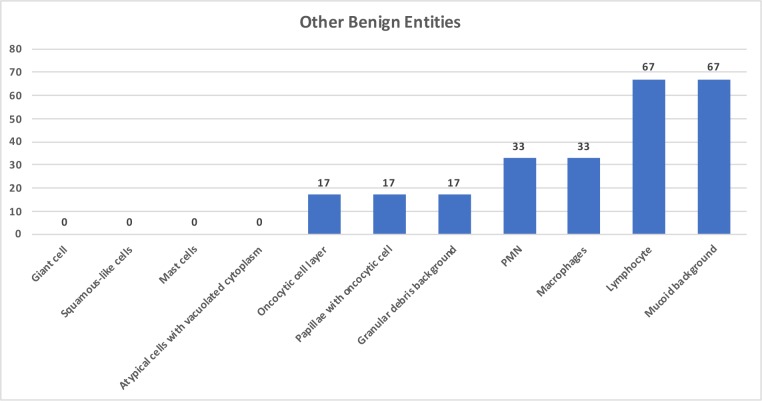
Graphic form of other benign entities cytomorphologic features as percentage.

In the 6 cases of other benign entities, cytomorphologic features were noted as lymphocytes in 4 (67%), mucoid background in 4 (67%), PMN cells in 2 (33%), macrophages in 2 (33%), oncocytic cell layer in 1 (17%), and papillae with oncocytic cells in 1 (17%) (Figure 2).

**Figure 3:**
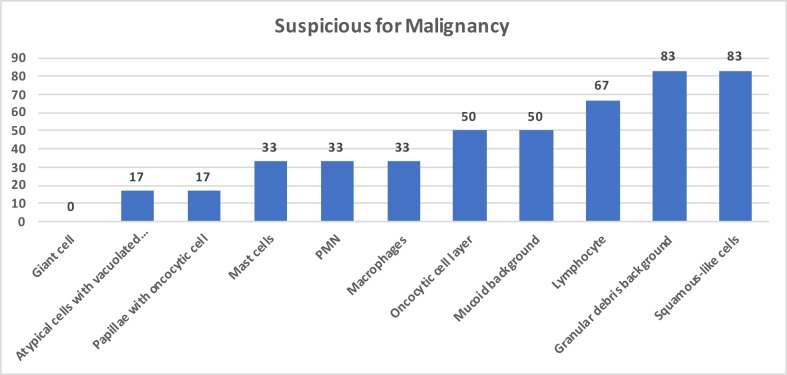
Graphic form of suspicious for malignancy cytomorphologic features as percentage.

Cytomorphologically we have seen squamous-like cells in 5 (83%), granular debris background in 5 (83%), lymphocytes in 4 (67%), oncocytic cell layer in 3 (50%), mucoid background in 3 (50%), PMN cells in 2 (33%), mast cells in 2 (33%), papillae with oncocytic cells in 1 (17%), and atypical cells with vacuolated cytoplasm in 1 (17%) case of the 6 cases suspicious for malignancy (Figure 3). Squamous-like cells were significant in the diagnosis of the group suspicious for malignancy (P = 0.005).

## 4. Discussion

WTs constitute nearly 5–10% of all salivary gland tumors and clinically present as painless masses unless infected. They are mostly seen in smoking male patients in the 5th and 6th decades [2,3,6–8]. WT is multicentric in 12–20% of patients and bilateral in 5–14% cases [2,3,7]. 

Pathogenesis of WT is not clear. It may originated from salivary duct inclusions in the lymph nodes [7] or may result from an inflammatory reaction [9]. Smokers show an increased tendency for WT. Retrograde flow of substances (or their excretion) from tobacco smoke into salivary ducts increases the risk of tumor formation [2,3,8–10].

Preoperative diagnostic accuracy of the solid and cystic lesions of salivary glands is high with FNAC. Flezar et al. and Syed et al. reported diagnostic accuracy for WTs as 95% and 74%, respectively [4,6]. Characteristic features of WTs in FNAC are lymphocytes and amorphous granular debris containing double rows of oncocytic cell layers [1,3–7,11,12] (Figure 4a). Various problems may cause misinterpretations of WTs in FNAC. The scarcity of typical features of the tumor or predominance of one of the main components seems to be related with misdiagnosis. The presence of inflammation and/or debris may obscure specimen cellularity (Figure 4b). Atypical and/or metaplastic features are other factors of misinterpretation [6,7,11–13].

**Figure 4a:**
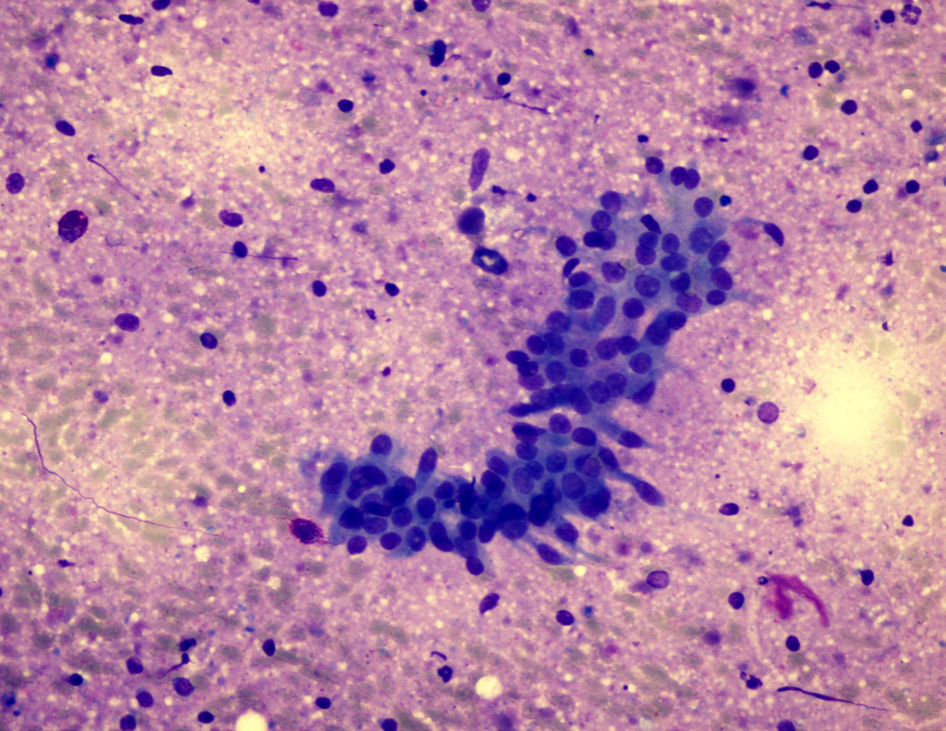
Characteristic features of WT in FNAC are lymphocytes and amorphous granular debris containing double rows of oncocytic cell layers (MGG, 400×).

**Figure 4b:**
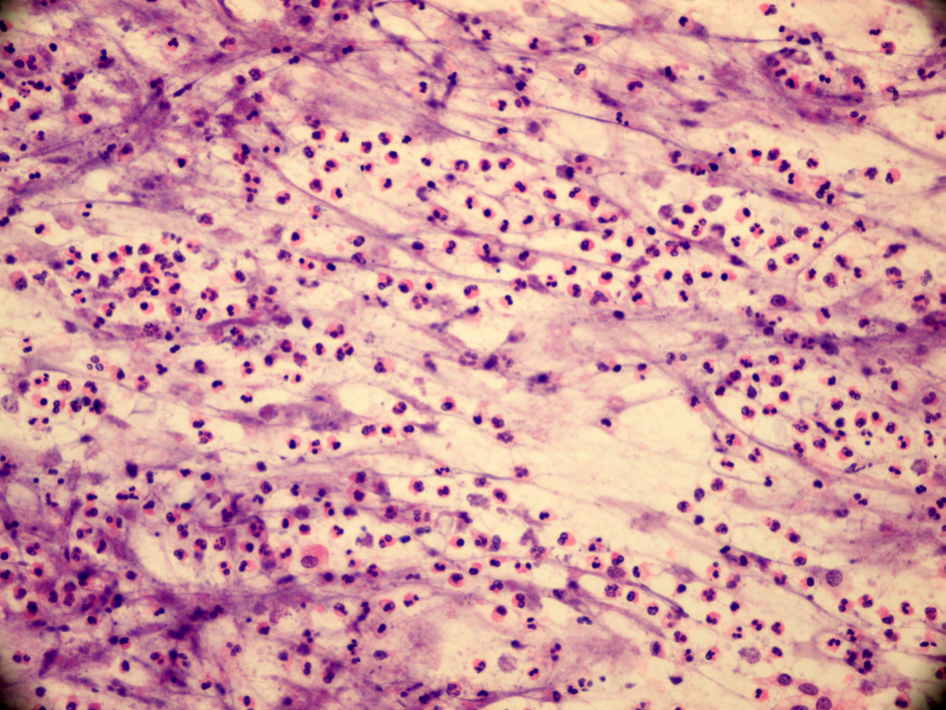
Dense inflammatory cells in mucoid background (MGG, 400×).

**Figure 4c:**
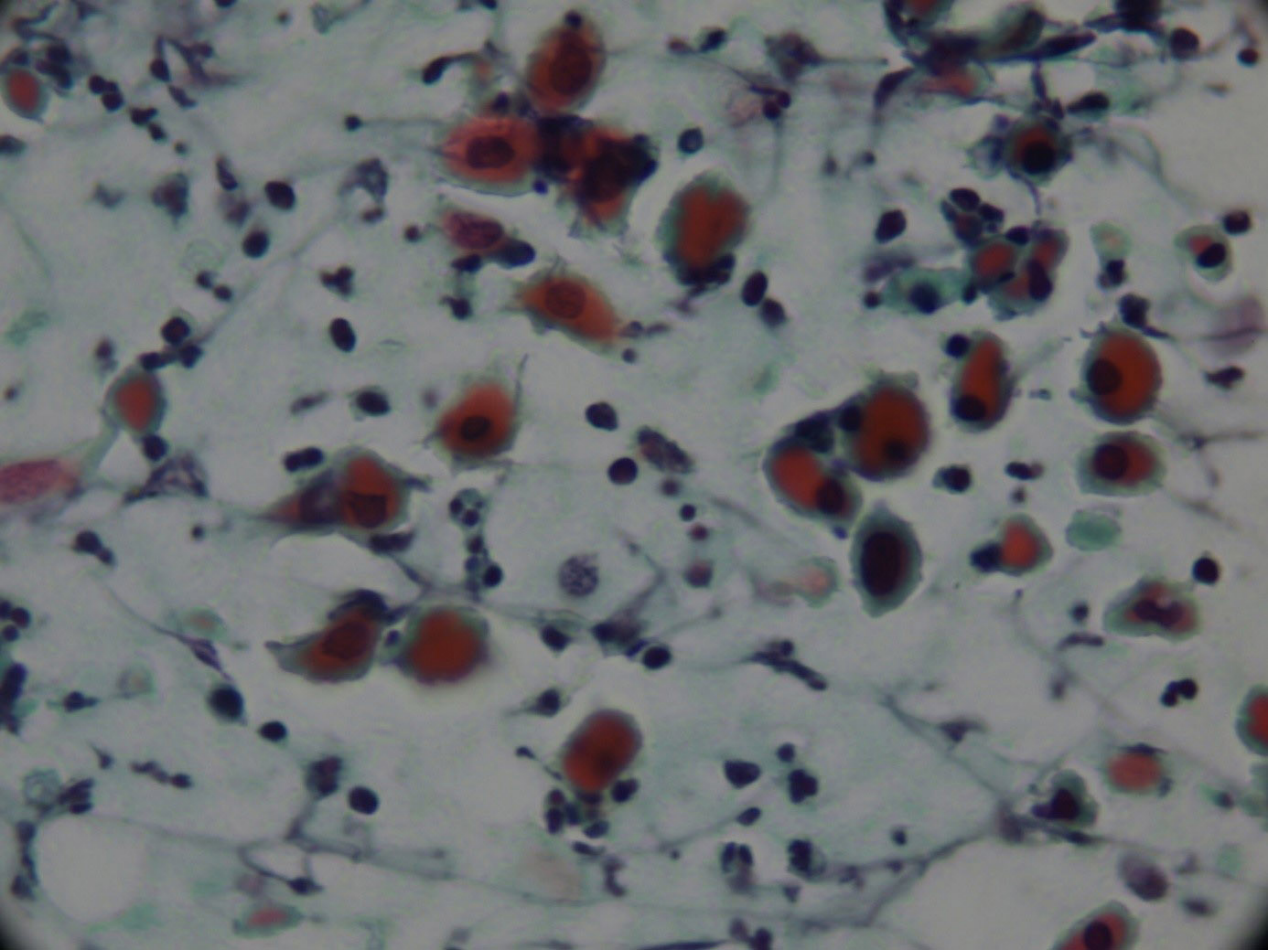
Degenerated oncocytic cells and squamous metaplastic cells (PAP, 400×).

**Figure 4d:**
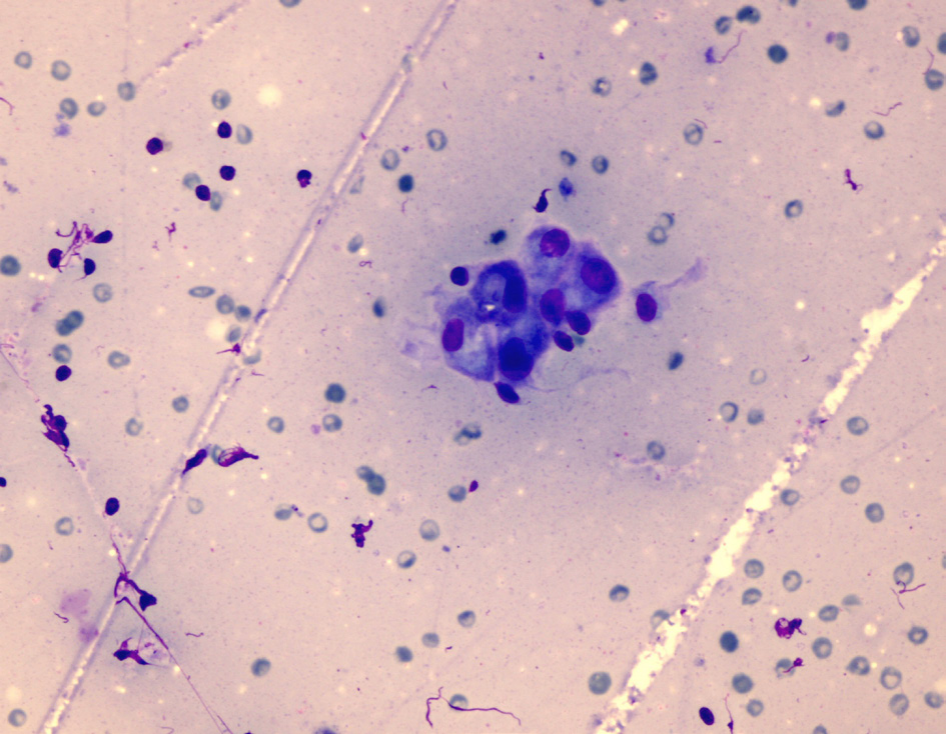
Atypical cell with vacuolated cytoplasm (MGG, 400×).

**Figure 4e:**
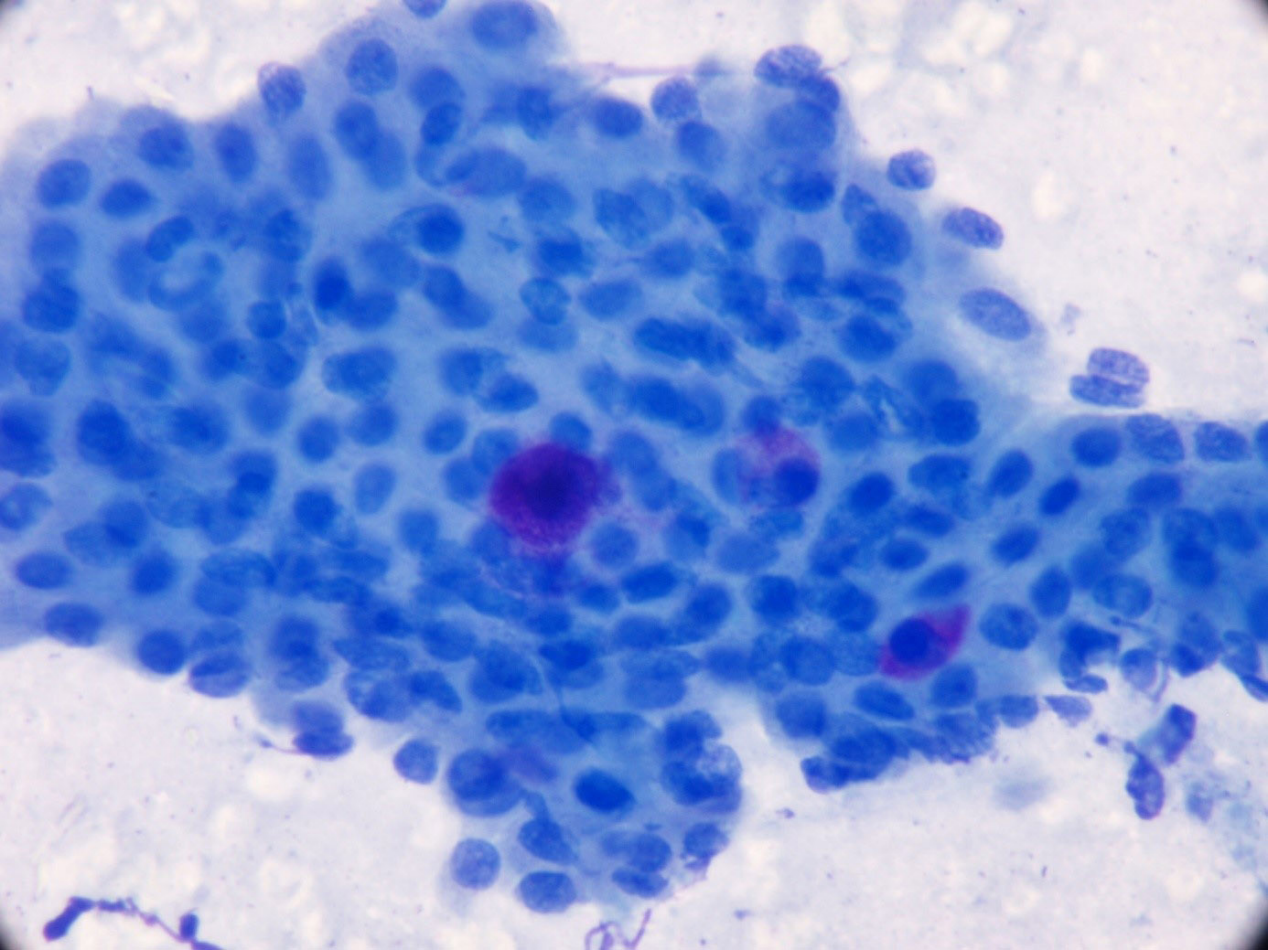
Mast cells together with oncocytic cell layer (MGG, 400×).

We observed 42 benign neoplasms and other benign entities and 6 cases suspicious for malignancy cytology in cytologic examination of 48 cases. In 36 of 42 cases in the benign neoplasms and other benign entities group, the cytologic diagnosis was WT. Characteristic criteria of these patients were lymphocytes in 33 (92%), oncocytic cell layer in 30 (83%), granular debris background in 24 (67%), mast cells in 23 (64%), papillae with oncocytic cells in 22 (61%), PMN cells in 20 (56%), and macrophages in 21 (58%) cases. Adequate parameters were sufficient for correct diagnosis in our study. Similar results were also achieved by Klijanienko et al. and Ballo et al. [13,14]. Two or three of the diagnostic criteria have been found in 83% of the series of Klijanienko et al. [13]. Ballo et al. showed typical cytological findings in 81% of their 30 cases [14]. At least 2 of 3 WT patients had the main cytomorphologic features, i.e. lymphocytes, oncocytic cell layer, and granular debris background. When the 2 parameters were taken into account, this percentage was 83% in our study. Although lymphocytes, an important diagnostic criterion in WT, were found to be high in the benign neoplasm group, they did not reach the statistical significance rate. As lymphocytes are also seen in inflammatory lesions, this may be a reason for the insignificance.

 Among the 6 patients in the other benign entities group, there was one cytomorphologic diagnosis each of oncocytoma, chronic sialadenitis, inflamed cyst, and mucocele, and 2 diagnoses of intraparotid lymphoid tissue. 

Oncocytes are typical cell components of WT but excessive presence of oncocytic cells in the field may lead to a diagnostic mistake such as oncocytoma [15–17]. Werma et al. reported that oncocytic epithelial cells are more often seen as papillary fragments, sheets, acini, and isolated appearances in cases of oncocytoma. However, oncocytes in WT show sheets with minimal pleomorphism and centrally located nuclei [17]. Mukunyadzi et al. indicated that oncocytic cell clusters are small and flat in WT, but these cell groups are 3-dimensional clusters in oncocytoma and oncocytic carcinoma [3].

The presence of acini clusters associated with lymphocytes and PMN cells was interpreted as chronic sialadenitis and inflamed cyst in cytologic examination of the benign cytologic group. Viguer et al. noticed that if aspiration was cystic and had no oncocytic cells, it could be diagnosed as benign nonspecific salivary gland cyst [11]. 

The case diagnosed as mucocele displayed an obvious mucoid background so could not be reported as WT in the cytologic examination. In cystic salivary gland tumors the cellularity of aspirates is poor and in these tumors insufficient sampling and false negative results are more prominent [5]. 

 The two cases diagnosed as intraparotid lymphoid tissue displayed lymphocytes with various stages of maturation and were reported as lymph nodes cytologically*. *Plasmocytes, mast cells, macrophages, and B lymphocytes constitute the lymphoid stroma in WT [7]. In addition, even reactive follicular germinal center elements may be seen [3]. Viguer et al. reported that an aspirate dominated by lymphoid component could not be distinguished from the reactive lymph node [11]. 

Cytomorphologic findings of 6 cases cytologically suspicious for malignancy had squamous-like cells in 5 (83%), granular debris background in 5 (83%), lymphocytes in 4 (67%), oncocytic cell layer in 3 (50%), mucoid background in 3 (50%), PMN cells in 2 (33%), mast cells in 2 (33%), atypical cells with vacuolated cytoplasm in 1 (17%), and papillae with oncocytic cells in 1 (17%). The presence of squamous or mucoid metaplasia and/or mucoid background are cytological difficulties responsible for misdiagnosis and should be differentiated from primary salivary gland squamous cell carcinoma, mucoepidermoid carcinoma, intraparotid metastasis from head and neck squamous cell carcinoma, and squamous cell carcinoma or mucoepidermoid carcinoma (MEC) that originated from WT [10,21,22]. Sood et al. have stated that diagnostic problems are common in cases that have intermediate-like cells and atypical squamous cells in the mucoid background [7]. For this reason, squamous cells should be interpreted carefully and other cytomorphologic components together with the clinical picture need to be considered to give a precise diagnosis. The majority of the squamous-like cells are not real squamous cells but are commonly degenerative and apoptotic oncocytes [11]. 

Degenerative oncocytes are common in the epithelial layer and spill into the fluid of the lumen. This condition commonly occurs in WT and indicates the apoptosis of oncocytes in the luminal fluid [7,11], and these degenerated oncocytes may resemble squamous cells [16]. The percentages of degenerated oncocytic cells were 65% and 66% in the studies of Flezar et al. and Viguer et al. [4,11]. In our study, we found squamous-like cells in 83% of the suspicious cytology group, in 25% of the WT group, and none in the benign cytology group (Figures 1–3, 4c). 

Benign signet ring cells may be seen in benign salivary gland tumors, and the origins of these cells are salivary gland excretory or striated duct basal cells. They may be differentiated by their capacity for mucus secretion [22]. In one case of our study, we detected an atypical cell with vacuolated cytoplasm (Figure 4d). Bellevicine et al. pointed out that existence of signet ring cells together with oncocytes supports the diagnosis of WT [22]. 

Mast cells are bone marrow-derived inflammatory cells characterized by their remarkable cytoplasmic granules. These cells are present in all human tissues (excluding avascular tissues such as bone and cartilage) and are in association with connective tissue structures [18,19]. Ashkavandi et al. showed that mast cells accumulated around the salivary gland tumors, and in all the tumoral samples the peritumoral area presented significantly higher mast cells than the intratumoral stroma. They did not find any difference in the number of mast cells between benign and malignant salivary gland tumors [18]. Flezar et al. noted coexistence of oncocytic cell layers with degranulated or degenerated cytoplasm mast cells in 79% of the WT cases [4]. Bottles et al. saw single-layer oncocytic cells with mast cells in 80% of their WT cases and recorded that there were no or seldom mast cells in MEC, pleomorphic adenoma, acinic cell carcinoma, and squamous cell carcinoma patients [20]. In 23 (64%) of WT cases of our study, we have seen mast cells stained by MGG in smears, and this was statistically significant (Figure 4e). We believe that the presence of localized mast cells together with an oncocytic cell layer should also be included as typical cytomorphologic findings of WT as remarked in the literature [4,10,18–20]. 

In conclusion, an experienced (cyto)pathologist does not have any difficulty in the diagnosis of WT, but the absence or lack of characteristic features with or without the presence of squamous-like cells, vacuolated cytoplasmic cells, and inflammatory reaction may cause diagnostic dilemma. Presence of mast cells is supplementary to avoid potential diagnostic failures. In any suspicious situation, it is rational not to make a specific WT diagnosis.
